# Digital Public Health in Deutschland: Status quo, Herausforderungen und Zukunftsperspektiven: Ein Positionspapier des Fachbereichs Digital Public Health der Deutschen Gesellschaft für Public Health e. V.

**DOI:** 10.1007/s00103-024-03989-0

**Published:** 2024-12-05

**Authors:** Laura Maaß, Christoph Dockweiler, Zora Hocke-Bolte, Stephanie Hoffmann, Florian Fischer, Sarah Forberger, Janika Gebert, Felix Holl, Robert Hrynyschyn, Sven Kernebeck, Claudia Pischke, Jacqueline Posselt, Jacob Spallek

**Affiliations:** 1 Universität Bremen, SOCIUM Forschungszentrum Ungleichheit und Sozialpolitik, Mary-Somerville-Straße 3, 28359 Bremen, Deutschland; 2 Leibniz ScienceCampus Digital Public Health Bremen, Bremen, Deutschland; 3 Fachbereich Digital Public Health, Deutsche Gesellschaft für Public Health e. V., Berlin, Deutschland; 4 Department Digitale Gesundheitswissenschaften und Biomedizin, Universität Siegen, Siegen, Deutschland; 5 Department Gesundheitswissenschaften, HAW Hamburg, Hamburg, Deutschland; 6 Fachgebiet Gesundheitswissenschaften, Brandenburgische TU Cottbus-Senftenberg, Senftenberg, Deutschland; 7 Lausitzer Zentrum für Digital Public Health, Institut für Gesundheit, Brandenburgische TU Cottbus-Senftenberg, Senftenberg, Deutschland; 8 Charité-Universitätsmedizin Berlin, corporate member of Freie Universität Berlin and Humboldt-Universität zu Berlin, Institut für Public Health, Berlin, Deutschland; 9 Bayerisches Zentrum Pflege Digital, Hochschule für angewandte Wissenschaften Kempten, Kempten, Deutschland; 10 Leibniz-Institut für Präventionsforschung und Epidemiologie – BIPS, Bremen, Deutschland; 11 Universität Bremen, Fachbereich 11 Human- und Gesundheitswissenschaften, Bremen, Deutschland; 12 Institut DigiHealth, Hochschule Neu-Ulm, Neu-Ulm, Deutschland; 13 Charité-Universitätsmedizin Berlin, corporate member of Freie Universität Berlin and Humboldt-Universität zu Berlin, Institut für Gesundheits- und Pflegewissenschaft, Berlin, Deutschland; 14 Fachbereich Gesundheit, FH Münster University of Applied Sciences, Münster, Deutschland; 15 Medizinische Fakultät, Centre for Health and Society, Institut für Medizinische Soziologie, Heinrich-Heine-Universität Düsseldorf, Düsseldorf, Deutschland; 16 Institut für Epidemiologie, Sozialmedizin und Gesundheitssystemforschung, Medizinische Hochschule Hannover, Hannover, Deutschland

**Keywords:** Digital Public Health, Gesundheitswissenschaften, Stellungnahme, Deutschland, Digitalisierung, Digital public health, Health sciences, Position paper, Germany, Digitalization

## Abstract

Dieses Positionspapier des Fachbereichs Digital Public Health der Deutschen Gesellschaft für Public Health e. V. definiert Digital Public Health (DiPH) und beschreibt deren Ziele sowie Potenziale. Ferner geht es auf die derzeitige Situation und Herausforderungen sowie Handlungsbedarfe in Deutschland ein. Der Schwerpunkt liegt hierbei auf der Darstellung der flächendeckenden Förderung der (digitalen) Gesundheitskompetenz, der Anwendung von DiPH in der Prävention, Gesundheitsförderung und Versorgung, dem Einsatz innovativer, präventiver Strategien zur Vorbeugung nichtübertragbarer Krankheiten und der Lehre von DiPH innerhalb von Public-Health-Studiengängen. Zudem werden notwendige Maßnahmen und Forderungen zur Stärkung von DiPH in Deutschland resümiert.

## Abstract (en)

This position paper from the Digital Public Health Section of the German Public Health Association defines digital public health (DiPH) and describes its goals and potential. It also addresses the current situation and challenges as well as the need for action in Germany. The focus here is on the presentation of the nationwide promotion of (digital) health literacy; the application of DiPH in prevention, health promotion, and healthcare; the use of innovative preventive strategies for the prevention of non-communicable diseases; and the teaching of DiPH in academic public health programs. Finally, relevant measures and demands to strengthen DiPH in Germany are summarized.

## 1 Der Fachbereich Digital Public Health der Deutschen Gesellschaft für Public Health e. V.

Die Arbeitsgruppe Electronic Public Health wurde 2016 als Handlungsfeld der Deutschen Gesellschaft für Public Health e. V. (DGPH) gegründet und 2023 als Fachbereich Digital Public Health (DiPH) institutionalisiert. Mit seinen Mitgliedern zielt der Fachbereich darauf ab, Handlungsfelder von DiPH zu definieren und zu kommunizieren, DiPH-Forschung in Deutschland voranzutreiben, Netzwerke zu initiieren, Kapazitäten und Kompetenzen in der DiPH-Lehre aufzubauen und Projekte zur Weiterentwicklung von DiPH zu unterstützen [1]. DiPH wird aus der Sicht der Mitglieder für die Public-Health-Forschung und -Praxis in Deutschland definiert und in bereits etablierte Begriffe wie Gesundheitstelematik, Electronic Health (eHealth), Mobile Health (mHealth) und Digital Health eingeordnet. Darauf aufbauend werden der Status quo von DiPH in Deutschland eingeordnet, aktuelle Engpässe und Herausforderungen benannt sowie vorliegende Handlungsbedarfe und Forderungen an die Forschung, Lehre und Politik in Deutschland aufgezeigt.

## 2 Was ist Digital Public Health?

Der Begriff DiPH wurde erstmals 2017 von Public Health England als Nutzung veränderter Methoden in Public Health mit neuen digitalen Konzepten und Instrumenten verwendet [2]. Bereits 1997 setzte die Weltgesundheitsorganisation (WHO) erste Impulse mit ihrer Definition der *Gesundheitstelematik *als zusammengesetzten Begriff für Aktivitäten, Dienste und Systeme mit Gesundheitsbezug, welche über Informations- und Kommunikationstechnologien (IKT) für die Gesundheitsförderung, Krankheitsbekämpfung, Gesundheitsversorgung, -forschung und die gesundheitsbezogene Bildung eingesetzt wurden [3]. Diese Definition wurde 2001 von Eysenbach zu *eHealth *weiterentwickelt und bezeichnet die Schnittstelle zwischen populationsbezogener Public Health und individuenzentrierter Medizininformatik. Hierbei werden IKT eingesetzt, um die Gesundheitsversorgung lokal bis global zu verbessern [4]. Während die spätere Bezeichnung *mHealth *laut der WHO (2011) auf den Einsatz mobiler Endgeräte für die medizinische und Public-Health-Praxis abzielte (jedoch mit Schwerpunkt auf dem Monitoring von Patient:innen; [5]), beschäftigt sich der derzeit dominierende Begriff Digital Health ausschließlich mit dem Einsatz von IKT im Gesundheitswesen, insbesondere im Kontext der personalisierten Medizin und der Telemedizin [6].

Es scheint, dass über die Jahre der voranschreitenden Digitalisierung der holistische und interdisziplinäre Ansatz von Public Health zunehmend aus der oben beschriebenen Terminologie verdrängt und der Fokus auf die Digitalisierung der Versorgung gelegt wurde. Nur mit einer begrifflich klaren, operativen und strategischen Integration in Forschung und Praxis kann der Mehrwert der Public-Health-Perspektive erneut in der Digitalisierung der Gesundheit verankert werden. In Anlehnung an Odone et al. (2019) und Wong et al. (2022) versteht der Fachbereich DiPH daher nicht als neues Themenfeld, sondern als system- und populationsorientierten Oberbegriff, der dieselben Ziele wie Public Health verfolgt und dabei den Einsatz und die Auswirkungen digitaler Technologien berücksichtigt [7, 8]. Als solches beinhaltet DiPH sowohl populationsbezogene eHealth- und mHealth-Anwendungen (wie auch Digital Health) als auch systembezogene Ansätze (z. B. die Telematikinfrastruktur als konkreter Anwendungsbereich der Gesundheitstelematik). Im Rahmen von DiPH sollen die essenziellen Public-Health-Funktionen erreicht werden [9]. Der Oberbegriff umfasst im Sinne des Health-in-All-Policies-(HiAP-)Ansatzes [10] die Gesundheitsversorgung (z. B. elektronische Patient:innenakten; ePA), Gesundheitsförderung und Prävention (z. B. Gesundheits-Apps) sowie das (inter-)nationale Gesundheitsmonitoring und digitale Epidemiologie [11]. Ferner beleuchtet DiPH Folgen der Technologisierung in multidisziplinärer Perspektive (sozial, ethisch, versorgungspraktisch und gesundheitlich) und gestaltet die gesundheitspolitischen Rahmenbedingungen aktiv mit [10].

## 3 Status quo: Digital Public Health in Deutschland

Eine zentrale Herausforderung für DiPH besteht darin, zu verhindern, dass durch den Einsatz dieser Maßnahmen die gesundheitliche Ungleichheit verstärkt und die gesundheitliche Chancengleichheit erschwert wird. Hiervon sind besonders vulnerable Gruppen betroffen, wie beispielsweise Menschen im hohen Alter, mit geringem Einkommen, geringer Bildung, eingeschränkten Sprachkenntnissen und geringer (digitaler) Gesundheitskompetenz. Sie sind überdurchschnittlich oft benachteiligt im Zugang zu digitalen Gesundheitsangeboten und haben Schwierigkeiten, wichtige (digitale) Informationen zu verstehen, kritisch zu bewerten und Angebote gewinnbringend für die eigene Gesundheit zu nutzen [12]. Umso wichtiger ist es, diese Gruppen bei der Entwicklung von neuen Maßnahmen zu berücksichtigen.

DiPH steckt in Deutschland derzeit in der Lehre und in der Forschung zu großen Teilen in den Kinderschuhen. Während eher klinisch orientierte digitale Gesundheitsanwendungen (DiGA) deutlich an Fahrt aufnehmen und internationale Anerkennung erhalten [13, 14], bleiben bekannte Herausforderungen wie die digitale Spaltung der Gesellschaft, mangelnde (digitale) Gesundheitskompetenz [12] oder der Fokus auf der medizinischen Versorgung (entsprechend dem Digital-Health-Ansatz) bestehen.

Nachfolgend werden daher exemplarisch Chancen und Zukunftsperspektiven von DiPH anhand dreier Themen skizziert. Zusätzlich soll auf das derzeitige Lehrangebot von DiPH eingegangen werden.

### 3.1 Digitale Gesundheitskompetenz kann Patient:innenautonomie stärken und digitale Spaltung vermeiden

Die gesundheitliche Chancengleichheit in der Bevölkerung ist ein zentrales Anliegen von Public Health: Menschen sollen unabhängig von ihren Eigenschaften und Voraussetzungen über die gleichen Chancen verfügen, um individuell eine bestmögliche Gesundheit zu erreichen. Durch die Digitalisierung besteht das Risiko, (gesundheitsbezogene) Ungleichheiten fortzusetzen oder zu verstärken [15]. Als digitale gesundheitliche Spaltung (Digital Health Divide) wird die ungleiche Verteilung des Zugangs zu und der Nutzung von (Gesundheits‑)Informationstechnologien in der Bevölkerung bezeichnet, welche sich wiederum auf die Inanspruchnahme und Wirksamkeit von DiPH-Maßnahmen auswirken kann [16].

Obwohl diese Herausforderung umfassend bekannt ist, sind die Maßnahmen zur Verringerung in der deutschen Public-Health-Praxis nicht flächendeckend implementiert. Circa 96 % aller Haushalte waren im Jahr 2022 an das Internet angeschlossen, 92 % hatten einen eigenen Computer oder Laptop und 88 % ein Smartphone [17]. Hinsichtlich der Nutzungszahlen waren im Jahr 2023 ca. 5 % der Bevölkerung zwischen 16–74 Jahren (3,1 Mio. Menschen) noch nie online [18], was 1 % weniger ist als im Vorjahr (6 % bzw. 3,4 Mio. Menschen; [19]). Trotz eines zunehmenden Angebots an digitalen Diensten in allen Lebensbereichen besteht somit weiterhin die Gefahr, vulnerable Gruppen abzuhängen [20]. Hieraus folgt, dass sich DiPH an einer fragilen Stelle zwischen der Verstärkung und Reduzierung von Ungleichheiten als Folge der Technologienutzung befindet. Um Risiken zu verringern und Potenziale auszuschöpfen, bedarf es über den Zugang hinaus der systematischen Stärkung gesundheits- und digitalbezogener Kompetenzen von Bürger:innen und Institutionen. Diese bildet die Grundlage für eine informierte Nutzung digitaler Technologien und Angebote und trägt dazu bei, digitale Spaltungen zu reduzieren [21].

Gleichzeitig bietet DiPH die Chance, gesundheitliche Ungleichheit zu verringern und den Zugang zu evidenzbasierter Gesundheitsversorgung und -information zu verbessern [22]. Am Beispiel von Online-Konsultationen zeigt sich, dass dieses Potenzial nicht ausgeschöpft wird. So erreichte die Zahl der gesetzlich abgerechneten Videosprechstunden seit 2019 ihren Höchststand im Jahr 2021 mit 3,5 Mio. abgerechneten Videosprechstunden. Bereits im Folgejahr waren es aber nur noch 2,7 Mio. [23], obwohl digitale Medien einen schnellen und einfachen Zugang zu relevanten und qualitätsgesicherten Gesundheitsinformationen bieten können. Voraussetzung ist, dass diese Angebote für die gesamte Bevölkerung im gleichen Maße zugänglich und geeignet sind [24]. Ebenso können digitale Medien und Anwendungen dazu beitragen, Präventions- und Gesundheitsangebote zu optimieren und an die Bedürfnisse von Zielgruppen anzupassen oder Bevölkerungsgruppen zu erreichen, die traditionelle Präventions- und Gesundheitsangebote beispielsweise durch sprachliche, räumliche oder zeitliche Restriktionen nicht in Anspruch nehmen [16].

Interaktive Plattformen sind in der Lage, das Mitwirken von Patient:innen an der eigenen Versorgung zu stärken. Beispiele für partizipative Plattformen sind in Deutschland rar. Nebolus, eine digitale Intervention zur Steigerung der Gesundheitskompetenz, ist zurzeit eines von nur wenigen staatlich geförderten Projekten mit partizipativen Ansätzen [25]. Angesichts nicht immer ausreichend ausgeprägter (digitaler) Gesundheitskompetenz sind derartige Ansätze besonders relevant [26]. Solche Angebote können dazu beitragen, dass Nutzende besser informierte Entscheidungen über ihre Gesundheit treffen können, wodurch die Qualität der Gesundheitsversorgung verbessert werden kann [16, 27].

### 3.2 Politik und Recht fokussieren klinische Gesundheitsversorgung

Im Koalitionsvertrag hat die Bundesregierung im Dezember 2021 die digitale Transformation des Gesundheitssystems zu einer Priorität erklärt [28]. Hierzu wurden in der Digitalisierungsstrategie unter anderem Ziele zur Weiterentwicklung der Telematikinfrastruktur, der ePA, dem elektronischen Rezept sowie zur Interoperabilität definiert [29]. Zudem wurden die nutzer:innenorientierte Neuausrichtung der Gematik als digitale Gesundheitsagentur sowie die bessere Nutzbarkeit von Versorgungsdaten für Forschungszwecke adressiert [30].

Vordergründig beschränken sich die digitalisierungsbezogenen, gesundheitspolitischen Initiativen auf die klinische Versorgung, sodass beispielsweise im Präventionsgesetz DiPH als Handlungsfeld nicht aufgegriffen wird [31]. Im Gegensatz bezieht sich die deutsche Digitalisierungsstrategie zwar auch auf den Gesundheitssektor, fokussiert jedoch fast ausschließlich den Versorgungsbereich und die ePA. Andere Bereiche, wie beispielsweise die Prävention und Gesundheitsförderung, werden durch die Strategie nicht abgedeckt [29]. Ebenso fehlt es am Einbezug weiterer Politikfelder, um DiPH im Sinne von HiAP zu praktizieren. Exemplarisch könnten die Integration von Klima- und Verbraucher:innenschutz, die Aus- und Weiterbildung der benötigten Expert:innen (siehe Abschn. 3.4) und Anwender:innen oder eine digitale Präventionsstrategie weitere Ansatzpunkte für DiPH bilden.

Public Health fordert in Deutschland bereits seit Jahren die Einbindung von Präventionsansätzen in die Gesundheitsversorgung. Das neue Bundesinstitut für öffentliche Gesundheit (BIÖG, Stand Oktober 2024) wird beweisen müssen, dass es dies besonders in den nichtklinischen Bereichen von Public Health leisten kann. Vor dem Hintergrund neuester Analysen zeigt sich, dass ein solcher Ansatz in Nachbarländern erfolgreich ist und sowohl die Lebenserwartung erhöhen als auch Ausgaben einsparen kann [32]. Neben einem stärkeren Fokus auf digitale Präventionsangebote müssen auch deren Implementierung, Akzeptanz und Adhärenz Berücksichtigung finden. DiPH kann für die Evaluation und Implementierung digitaler Interventionen ausreichende und belastbare Daten bereitstellen und Tools für ein kosteneffizientes Erreichen der Public-Health-Ziele bieten [33–35]. Hierdurch können die Passgenauigkeit, Effizienz, Geschwindigkeit und Legitimität von gesundheitsfördernden und präventiven Maßnahmen erhöht werden, während zeitgleich Probleme schneller identifiziert und Lösungen passgenauer konzipiert werden können, um bestehende Techniken zu ergänzen [36]. Auch in diesem Kontext ist die Implementierung auf die klinische Gesundheitsversorgung fokussiert, sodass derzeit keine rechtliche Grundlage für die Einbindung von digitalen Diensten wie DiGA (§ 139e SGB V) und digitalen Pflegeanwendungen (DiPA) nach § 78a SGB XI für die Primärprävention besteht.

Im Strategiepapier „Eckpunkte einer Public-Health-Strategie für Deutschland“ des Zukunftsforums Public Health wurden die Möglichkeiten von DiPH für den Ausbau und die Weiterentwicklung von Surveillance und Gesundheitskommunikation thematisiert, grobe Forschungslinien der Zukunft im Rahmen von digitaler Transformation skizziert (z. B. die Analyse von Social-Media-Inhalten sowie der Umgang mit Big Data oder künstlicher Intelligenz; KI) und auf eine erforderliche Weiterentwicklung von Forschungsmethoden in diesem Bereich hingewiesen [37]. Diese Skizzen müssen nun über eine entsprechende Institutionalisierung in den Praxis- und Forschungsalltag integriert werden.

**Abb. 1:**
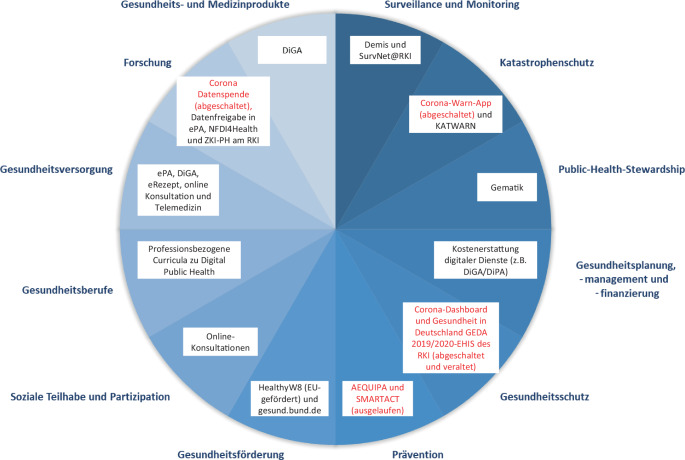
Digitale Public-Health-Interventionen in Deutschland entlang der essenziellen Public-Health-Funktionen. Eigene Darstellung nach WHO 2024 [38]. Legende: *DEMIS* Deutsches Elektronisches Melde- und Informationssystem für den Infektionsschutz, *DiGA* digitale Gesundheitsanwendung, *DiPA* digitale Pflegeanwendung, *EHIS* European Health Interview Survey, *ePA* elektronische Patient:innenakte, *GEDA* Gesundheit in Deutschland aktuell, *Gematik* Nationale Agentur für Digitale Medizin, *NFDI4Health* Nationale Forschungsdateninfrastruktur für personenbezogene Gesundheitsdaten, *RKI* Robert Koch-Institut, *ZKI-PH* Zentrum für Künstliche Intelligenz in der Public-Health-Forschung, *rote Farbcodierung* Projekt ausgelaufen bzw. Dienst abgeschaltet

In Abb. 1 werden die aktualisierten essenziellen Public-Health-Funktionen der WHO [38] mit DiPH-Ansätzen aus Deutschland exemplarisch ergänzt, welche derzeit auf nationaler Ebene implementiert sind bzw. waren. Bewusst werden kleinere lokale Pilotprojekte sowie Forschungsprojekte nicht berücksichtigt, um den Fokus auf die Bundesebene zu lenken. Die Abbildung verdeutlicht, dass derzeit auf nationaler Ebene nicht alle geforderten Funktionen gewährleistet werden oder zum Teil Strukturen aus der COVID-19-Pandemie (2020–2022) zurückgebaut wurden, wie in Rot dargestellt. Aus der öffentlichen Hand finanzierte und implementierte Interventionen zur Primärprävention beschränkten sich größtenteils auf vom Bundesministerium für Bildung und Forschung geförderte Forschungsverbünde wie AEQUIPA [39] und SMARTACT [40], die jedoch ebenfalls mittlerweile ausgelaufen sind. Gleichzeitig zeigt sich auch hier deutlich der Fokus auf Ansätzen in der klinischen Versorgung mit dem Ausbau der ePA und DiGA.

Die Analyse zeigt darüber hinaus, dass in den Bereichen Katastrophen- und Gesundheitsschutz sowie Prävention Handlungsbedarfe und zugleich ungenutzte Potenziale bestehen. Die Vielzahl ausgelaufener Ansätze lässt darauf schließen, dass diese nicht ausreichend nachhaltig im System verankert waren. Eine Überleitung der initialen Pilotförderung hin zu nachhaltig etablierten Systemen muss gelingen, um limitierte Ressourcen bestmöglich einzusetzen. Ferner sollte die Übertragbarkeit von themenspezifischen Interventionen (beispielsweise Tools mit Bezug zur COVID-19-Pandemie) auf andere Systemprobleme bzw. meldepflichtige Infektionserkrankungen überprüft und gestärkt werden.

### 3.3 Potenzial präventiver digitaler Strategien bei chronischen Erkrankungen

Nichtübertragbare Erkrankungen wie Herz-Kreislauf-Erkrankungen, Krebs, chronische Atemwegserkrankungen und Diabetes mellitus stellen auch in Deutschland eine erhebliche Herausforderung für die öffentliche Gesundheit dar. Hierzulande tragen diese Erkrankungen zu 91 % aller Todesfälle bei [41]. DiPH kann dieser Krankheitslast mit präventiven und gesundheitsfördernden Strategien entgegenwirken, indem sie sowohl die Verhaltens- als auch die Verhältnisebene adressiert.

Über die Einbindung sozialer Medien kann gesundheitsrelevanter Inhalt von Betroffenen direkt aus der „digitalen Lebenswelt“ identifiziert und genutzt werden, um Bedarfe zu analysieren und daraus Strategien für Gesundheitsakteure abzuleiten [42]. In der bisherigen Forschung erfolgte die Anwendung neuer Methoden und interventioneller Ansätze jedoch häufig professions- oder disziplinenspezifisch, sollte aber im Rahmen von DiPH stärker verflochten werden. Durch den weitreichenden Einsatz von KI können Interventionen noch stärker auf Individuen und Kontexte maßgeschneidert und ihre Auswirkungen potenziert werden. Ebenso können Analysen von groß angelegten Datensammlungen durchgeführt und dadurch Präventionsansätze identifiziert werden [43].

Internationale positive Ergebnisse einer solchen interdisziplinären Zusammenarbeit sind z. B. KI-basierte Interventionen zur Steigerung von körperlicher Aktivität [44], die Technografie [45], räumliche Mikrosimulationen [46], Foresight-Studien [47] oder der Einsatz von Mobile Experience Sampling Tools [48]. Für Deutschland erscheint die Interventionslandschaft jedoch eher karg. Bekannter hingegen ist hierzulande die Anwendung von Big Data in der Gesundheitsförderung und Prävention [49]. Dazu zählen Digital Epidemiology [11], Data Mining oder Geosocial-Data-Analysen [50]. Diese Forschungsbereiche müssen über interdisziplinäre Kooperationen vertieft und ausgebaut werden, um eine ausreichende Grundlage für die evidenzbasierte Entwicklung digitaler Strategien in der Gesundheitsversorgung und in der Prävention und Gesundheitsförderung bereitzustellen.

### 3.4 Akademische Lehre in Deutschland: Digital Public Health dringend gesucht

Seit fast 30 Jahren existiert in Deutschland eine Vielfalt verschiedener akademischer Aus‑, Fort- und Weiterbildungsangebote, die in unterschiedlicher Granularität Public-Health-Inhalte adressieren. Um die für Gesundheit und Krankheit wesentlichen Prozesse der digitalen Transformation zu analysieren, zu verstehen, anhand einer sozialgerechten Reflexion zu begleiten und diese auch mit Blick auf eine für Menschen bedeutsame, handhabbare und verständliche Strukturentwicklung zu gestalten, bedarf es einer dringenden Erweiterung bestehender Qualifikationsziele in der Public-Health-Ausbildung in Deutschland. Zu den notwendigen Qualifikationen zählen unter anderem das sichere Beherrschen von IT-Anwendungen zur Datenanalyse und -aufbereitung, das Verständnis ethischer und rechtlicher Anforderungen in der Verwendung und Verarbeitung digital erfasster Gesundheitsdaten, die Nutzungskompetenz von digitalen Anwendungen in den Kernbereichen von Public Health sowie die erforderliche Sensibilisierung für kulturelle Einflüsse auf den Einsatz digitaler Technologien in der Versorgung [51]. Technikfolgen müssen unter sozialer, versorgungspraktischer und bevölkerungsgesundheitlicher Hinsicht reflektiert werden können, um zukünftig (gesundheits-)politische Rahmenbedingungen mitzugestalten.

Eine Analyse des Fachbereichs DiPH konnte eindrücklich aufzeigen, dass hierfür in Deutschland derzeit kein ausreichendes Lehrangebot besteht [52]. Lediglich 16 der 79 Studiengänge, die sich weitestgehend an den Public-Health-Kernkompetenzen der Association of Schools of Public Health in the European Region (ASPHER) orientierten, zeigen curriculare Bezüge in den Lehrinhalten und Kompetenzzielen zu DiPH auf. Die Mehrzahl dieser Studiengänge (*n* = 12) wird in Deutschland an Hochschulen angeboten. Lediglich 4 universitäre Studiengänge bieten aktuell eine Kompetenzvermittlung im Bereich DiPH an. In der Mehrzahl der Studiengänge werden die Inhalte als Pflichtbereiche gelehrt, zeigen sich mit Blick auf die Gewichtung der Handlungsfelder von Public Health allerdings inhaltlich sehr heterogen [53]. Die Handlungsfelder Surveillance, Krisenplanung und -reaktion sowie Governance sind mit ihren Bezügen zur Digitalisierung derzeit unzureichend bis gar nicht in der Ausbildung adressiert [52].

## 4 Forderungen zur Stärkung von Digital Public Health in Deutschland

Die nachstehenden Forderungen zielen auf eine Stärkung der derzeitigen Forschung, Praxis, Institutionalisierung und Lehre von DiPH ab, um die oben exemplarisch aufgezeigten Herausforderungen in Deutschland zu überwinden. Sie adressieren hierbei Forschende, Bildungseinrichtungen und die Politik in Deutschland.

### 4.1 Forschung und Praxis

#### 4.1.1. Anwendungsmöglichkeiten und Entwicklung von Digital-Public-Health-Methoden in Deutschland

- **Innovative Methoden und Technologien einsetzen: **Um den Herausforderungen der **Digitalisierung** im Kontext von Public Health zu begegnen, muss eine deutliche Stärkung der DiPH-Forschungslandschaft in Deutschland erfolgen. Hierzu ist die Forschung zur Entwicklung von neuen Methoden erforderlich, die eine bessere Einbeziehung von Nutzer:innen mit partizipativen und kokreativen Ansätzen ermöglichen. Außerdem gilt es, die Anwendungsmöglichkeiten und den Nutzen von innovativen Technologien (generativer) KI zur Analyse großer Datenmengen und Datenformen (Big Data, Digital Epidemiology) für DiPH auszuschöpfen.
- **Digital Divide im Kontext Gesundheit reduzieren:** Zur Überwindung der digitalen Spaltung und Identifikation von Ansatzpunkten für inkludierende DiPH-Maßnahmen ist weitere sozialepidemiologische Forschung über individuelle, soziale und kontextuelle Ursachen erforderlich. Diese Ansatzpunkte sollten einen verbesserten Zugang zu überprüften Gesundheitsinformationen, die Förderung der digitalen Gesundheitskompetenz und die Förderung der Inanspruchnahme, Individualisierung und Effektivität von Präventions- und Gesundheitsinterventionen beinhalten. Hierbei müssen Bürger:innen stärker partizipativ in Forschung und Entwicklung eingebunden werden. Auch die Balance zwischen Datensicherheit und Datenschutz bei der dynamischen Entwicklung von Maßnahmen gilt es auszutarieren.
- **Prädiktive Modelle entwickeln:** Die COVID-19-Pandemie hat verdeutlicht, dass eine vorausschauende Forschung erforderlich ist, um zukünftigen Gesundheitsrisiken mit digitalen Interventionen auf der Bevölkerungsebene zu begegnen. Hierbei ist die Durchführung von Prognosen und Szenarien zu berücksichtigen, um Nachhaltigkeit und Effizienz zu gewährleisten.
- **Nachhaltigkeit:** Digitalisierung darf kein Selbstzweck sein, sondern sollte auf die nachhaltige Lösung aktueller Probleme im Gesundheitswesen abzielen. Dazu zählen beispielsweise die Verminderung der Krankheitslast durch gesundheitsfördernde und präventive Strategien oder Chancen, dem Fachkräftemangel entgegenzuwirken.

#### 4.1.2. Interdisziplinarität und sektorübergreifende Zusammenarbeit

e) **Zusammenarbeit stärken:** Wir befürworten eine verstärkte interdisziplinäre und interprofessionelle, institutionsübergreifende sowie zielgerichtete Zusammenarbeit und die Einbindung verschiedener Stakeholder im Gesundheitswesen und darüber hinaus. Hierunter fallen Disziplinen wie die Pflege, Medizin, Informatik, Epidemiologie, (Gesundheits‑)Ökonomie, Ethik, Soziologie, Politikwissenschaften, Public Health, Psychologie, Kommunikationswissenschaften.
f) **Partizipative Ansätze einsetzen:** Es ist unabdingbar, die Stärkung von DiPH differenziert und bedarfsgerecht mithilfe von partizipativen Ansätzen zu gestalten. Digitalisierung sollte nicht aus reinem Trendbewusstsein erfolgen, sondern gezielt eingesetzt werden, um konkrete gesundheitsbezogene Herausforderungen zu erkennen, zu bewältigen und die Versorgung zu verbessern. Dies ist besonders deshalb relevant, um begrenzte Ressourcen effektiv und effizient einzusetzen.

### 4.2 Institutionalisierung und Internationalisierung

#### 4.2.1. Ziele und Aufgaben des BIÖG

g) **Bevölkerungsbezug im BIÖG umsetzen:** Das neu gegründete Bundesinstitut sollte DiPH umfassend adressieren. Hierbei muss der Bevölkerungsbezug im Mittelpunkt stehen. Dazu ist erforderlich, dass das BIÖG die oben genannten Aspekte zur Erforschung von (generativer) KI und Vermeidung des Digital Divide sowohl in der Erhebung von Gesundheitsdaten als auch in der Ableitung von präventiven und gesundheitsfördernden Maßnahmen adressiert.
h) **Zusammenführen von multidisziplinären Daten: **Die digitale Transformation hat Auswirkungen auf alle Bereiche des täglichen Lebens und folglich einen hohen Einfluss auf die Gesundheit der Bevölkerung. In diesem Zuge ist eine Zusammenarbeit aller Politikbereiche im Sinne des HiAP-Ansatzes erforderlich. Dies bedingt, dass Daten aus verschiedenen Quellen unter Einhaltung datenschutzrechtlicher und ethischer Vorkehrungen innerhalb und außerhalb des Gesundheitswesens miteinander verknüpft werden. Beispielsweise lässt sich dies an der gemeinsamen Betrachtung von Gesundheits‑, Klima- und Umweltdaten darstellen.

#### 4.2.2 Mehr Digital Public Health in Präventionsleitlinien und Digitalisierungsstrategien

i) **Digitalisierungsstrategie mit Präventions- und Gesundheitsförderungsgedanken: **Während die Digitalisierungsstrategie einen guten Anfang bietet, ist eine Weiterentwicklung dieser vor dem Hintergrund der Prävention und Gesundheitsförderung mit klar definierter Terminologie sowie Nutzer:innenorientierung, Transparenz und partizipativen Ansätzen als übergeordnetem Ziel der Strategie unabdingbar.

### 4.3 Lehre und Qualifizierung

#### 4.3.1 Stärkung von Digital Public Health in Aus‑, Fort- und Weiterbildung von Bürger:innen und Berufsgruppen abseits von Public Health

j) **Digitale Gesundheitskompetenz stärken:** DiPH muss in Aus- und Weiterbildungsprogramme von Gesundheitsprofessionen und dem Öffentlichem Gesundheitsdienst sowie bei der Bildung von Bürger:innen und Berufsgruppen abseits der klassischen Public-Health-Ausbildung integriert werden. Es ist hierbei an der Politik, entsprechende Strukturen zur Stärkung der digitalen Gesundheitskompetenz aufzubauen und auf ihre Wirksamkeit hin zu evaluieren.

#### 4.3.2 Stärkung von Digital Public Health in der akademischen Public-Health-Lehre

k) **Stärkere Orientierung an Bedarfen der Public-Health-Forschung und -Praxis: **Die digitale Transformation formt unsere Lebenswelten mit daraus resultierenden Implikationen für Gesundheit und Krankheit. Entsprechend fordert der Arbeitsmarkt zunehmend entsprechende Qualifikationen von Absolvent:innen. DiPH als essenzieller und transdisziplinärer Teilbereich von Public Health muss folglich in Public-Health-Curricula in Form von obligatorischen Bestandteilen eingeführt und gestärkt werden, um diesen Ansprüchen auch zukünftig zu genügen. Dafür sind entsprechende Ressourcen bereitzustellen.
l) **Einheitliche Qualifikationsrahmen schaffen: **Um eine deutschlandweit vergleichbare Public-Health-Ausbildung zu garantieren und dem Arbeitsmarkt gleichwertige Absolvent:innen zur Verfügung zu stellen, müssen gleiche Inhalte an verschiedenen Public-Health-Standorten gelehrt werden, welche sich an einem einheitlichen bedarfsorientierten und stetig aktualisierten DiPH-Qualifikationsrahmen orientieren. Hierfür erscheint ein integrativer Prozess notwendig, der unter Beteiligung von Lehrenden, Studierenden, Akteur:innen der Public-Health-Praxis und den relevanten Fachbereichen der wissenschaftlichen Fachgesellschaften die Entwicklung eines Qualifikationsrahmens für DiPH unter Beachtung bestehender Strukturen vorantreibt. So soll sichergestellt werden, dass Public-Health-Studierenden auf Bachelor‑, Master- und Promotionsniveau vergleichbare Kompetenzen vermittelt werden, die sowohl internationalen Standards als auch zukünftigen Anforderungen eines sich wandelnden Arbeitsmarktes entsprechen.

## 5 Fazit

Obwohl bereits im Jahr 2017 benannt, steckt DiPH in Deutschland noch immer in seinen Anfängen. Jedoch existieren vereinzelt Ansätze in der Forschung, Gesundheitsförderung, Versorgung und akademischen Public-Health-Lehre, welche in den kommenden Jahren weiter ausgebaut und nachhaltig implementiert werden müssen. Der Fachbereich Digital Public Health der DGPH e. V. begrüßt diesen Wandel und wird ihn aktiv mitgestalten.
